# Impact of Coronary Collateral Circulation on In-Hospital Death in Patients with Inferior ST Elevation Myocardial Infarction

**DOI:** 10.1155/2015/242686

**Published:** 2015-11-25

**Authors:** Baris Yaylak, Bernas Altintas, Huseyin Ede, Erkan Baysal, Sukru Akyuz, Onder Bilge, Utkan Sevuk, Guney Erdogan, Haci Ciftci

**Affiliations:** ^1^Department of Cardiology, Diyarbakir Gazi Yasargil Training and Research Hospital, 21010 Diyarbakır, Turkey; ^2^Department of Cardiology, Bozok University School of Medicine, Yozgat, Turkey; ^3^Department of Cardiology, Siyami Ersek Thoracic and Cardiovascular Surgery Training and Research Hospital, Istanbul, Turkey; ^4^Department of Cardiovascular Surgery, Diyarbakir Gazi Yasargil Training and Research Hospital, Diyarbakır, Turkey; ^5^Department of Cardiology, Ordu State Hospital, Ordu, Turkey

## Abstract

*Objectives*. Coronary collateral circulation (CCC) may limit the size of right ventricular (RV) infarcts but does not fully explain the relationship between CCC and clinical adverse events in patients with inferior STEMI. In this study, it was aimed to assess the relationship between preintervention angiographic evidence of CCC and clinical outcomes in patients with inferior STEMI who have undergone percutaneous coronary intervention.* Methods*. A total of 235 inferior STEMI patients who presented within the first 12 hours from the symptom onset were included. CCC to the right coronary artery (RCA) before angioplasty were angiographically assessed, establishing two groups: 147 (63%) patients without CCC and 88 (37%) with CCC according to presence of CCC.* Results*. RV infarction, complete atrioventricular block, VT/VF, cardiogenic shock, and in-hospital death were noted less frequently in patients with CCC than in those without CCC. Absence of CCC to RCA was found to be the independent predictor for in-hospital death among them (odds ratio 4.0, 95% CI 1.8–12.6; *p* = 0.03).* Conclusion*. Presence of angiographically detectable CCC was associated with better in-hospital outcomes including RV infarction, complete AV block, cardiogenic shock, and VT/VF in patients with inferior STEMI.

## 1. Introduction

Inferior ST elevation myocardial infarction (STEMI) accounts for 40–50% of all acute STEMI [1]. Inferior STEMI are usually considered as having a more favorable prognosis than anterior STEMI. It is important to note, however, that approximately 50% of patients suffering inferior STEMI will have complications or distinguishing features associated with an increased mortality that will considerably change an otherwise favorable prognosis. One of the most important reasons for this difference is the presence of right ventricular infarction (RVI) which may accompany inferior STEMI. Actually, compared to the left ventricle, the right ventricle has several characteristics protecting it from ischemia: a lower oxygen requirement due to its smaller muscle mass and workload, direct diffusion of oxygen from the right ventricular (RV) cavity, and a greater oxygen supply due to more extensive collaterals [2–4]. The latter, coronary collateral circulation (CCC) in patients with STEMI, a relevant protective role of collaterals has been observed by development of smaller infarct size, preservation of cardiac function after acute infarctions, and reduction in postinfarct ventricular dilatation [5]. However, the general impact of the CCC on mortality has not been well established [5]. CCC may limit the size of RV infarcts but do not fully explain the relationship between CCC and clinical outcomes in patients with inferior STEMI. This study assessed the relationship between preintervention angiographic evidence of CCC and clinical outcomes in patients with inferior STEMI who have undergone mechanical intervention within 12 hours of symptom onset.

## 2. Methods

### 2.1. Study Patients

This prospective study was conducted between January 2012 and June 2015. A total of 235 subjects with inferior STEMI who presented within 12 hours from the symptom onset were included in the study. Inferior STEMI was defined as chest pain that lasts more than >30 minutes with an ST segment elevation >1 mm in any two consecutive inferior leads.

All subjects have undergone an early coronary angiogram followed by primary percutaneous coronary intervention (PCI) upon their admission. Subjects with diagnostic coronary angiography showing complete occlusion of the right coronary artery (RCA) (thrombolysis in myocardial infarction (TIMI) flow grades 0-1) were included in the study. Left and right coronary angiograms were obtained before the attempted angioplasty of sufficient quality to assess the filling of RCA and side branches by collateral circulation. Exclusion criteria were venous graft-related infarcts, ungraded collateral flow due to technical reason, concurrent pericardial disease, previous RV dysfunction, previous LV systolic dysfunction (defined as previously measured left ventricular ejection fraction of <50%), chronic pulmonary disease, pulmonary hypertension, valvular heart disease (moderate to severe insufficiency and/or stenosis), acute pulmonary embolism, history of cardiac arrest before admission, and renal failure (serum creatinine level > 1.5 mg/dL on admission). Also, the patients were treated with percutaneous coronary intervention (PCI) on the left circumflex artery or the left anterior descending artery as the culprit vessels were excluded. Additionally, we excluded patients with coronary artery stenosis of >70% at the left circumflex artery and/or the left anterior descending artery. Informed consent of each subject and approval of the local ethics committee were obtained.

### 2.2. Coronary Angiography and Primary PCI

Coronary angiography was performed in 90 minutes following admission. All patients received dual antiplatelet therapy with aspirin and clopidogrel (600 mg) or ticagrelor (180 mg) loading dose. Preprocedural anticoagulation consisted of intravenous unfractionated heparin (35–70 IU/kg) in all cases. PCI with stent implantation was performed according to current guidelines [6]. The purpose of the primary PCI procedure was to obtain a residual stenosis of <20% in the infarct-related artery (IRA) by visual evaluation. A successful angiographic result was defined which is associated with TIMI grade-2-3 flow. An unsuccessful procedure was defined as a procedure resulting in TIMI grade 0 or 1 flow whatever the residual stenosis was. In patients treated with tirofiban, the agent was administrated after primary PCI in the coronary care unit. The systemic bolus of tirofiban was used according to operator's decision and continued for the following 12 hours accordingly. Thrombus aspiration catheter was not used. Flow in the RCA and major RV branch artery was determined before and after PCI and graded as previously described [7]. The site of occlusion of the right coronary artery was defined as proximal or distal on the basis of the origin of the major (>1 mm in diameter) RV branch.

### 2.3. Coronary Collateral Circulation

Collateral flow from the patent vessels to the infarct-related artery was graded using the classification developed by Rentrop et al. [8]. Two experienced cardiologists assessed the coronary angiograms in a blinded fashion and reached a consensus regarding the TIMI flow grade and the collateral flow grade. Inter- and intraobserver agreements of Rentrop collateral grades were determined from a random sample of 50 coronary angiograms (*k* values were 0.825 [interobserver] and 0.9155 [intraobserver], resp.; *p* < 0.001 for both analyses). Patients were divided into two groups based on presence of CCC as follows: inferior STEMI without CCC, patients without angiographic collateral filling of RCA or side branches (Rentrop 0) and inferior STEMI with CCC, and patients with angiographic collateral filling of the RCA or side branches (Rentrop 1, 2, or 3).

### 2.4. Echocardiography

Patients underwent standard two-dimensional echocardiography with a digital ultrasonic device system (iE33; Philips, Netherlands) immediately after PCI. RV dysfunction was defined according to the rules set by American Society of Echocardiography [9]. Echocardiographic evaluation of the RV function was completed by the assessment of right ventricular fractional area change (RVFAC), tricuspid annular plane systolic excursion (TAPSE), and right ventricular free-wall motion. Also, from the apical four-chamber view, the right ventricular free wall was divided into three segments and the motion of each segment was scored on a scale of 1 to 4 (1: normal, 2: hypokinetic, 3: akinetic, and 4: dyskinetic). The overall score for right ventricular free-wall motion was calculated as the average score for the segments. Modified Simpson's method was used to assess the left ventricular ejection fraction (LVEF).

### 2.5. In-Hospital Clinical Course

Adjunctive medical therapy followed the standards of the coronary care unit. The primary objective of this study was to examine in-hospital death. Secondary objectives were to examine the occurrence of RV infarction, complete AV block, cardiogenic shock, and VT/VF. Cardiogenic shock was characterized by hypotension (defined as systolic blood pressure below 90 mmHg lasting more than 15 minutes), clear lungs, and elevated right heart filling pressure (JVP) in association with signs of tissue hypoperfusion (cold extremities, cyanosis, oliguria, or altered mental status) which were not derived from severe left ventricular dysfunction and extracardiac causes.

### 2.6. Statistical Analysis

Statistical analysis was performed using SPSS 18.0 (SPSS Inc., Chicago, IL). The Kolmogorov-Smirnov test was used to evaluated whether the distribution of continuous variables was normal. Continuous variables were compared with Student's *t*-test or Mann-Whitney *U* test according to the distribution of the data. Categorical variables were compared with chi-square or Fisher's exact tests when appropriate. Continuous variables were presented as mean + SD whereas categorical variables as count and percentages. Multiple logistic regression analysis was used to assess the independent predictors of in-hospital death. Odds ratios were calculated with corresponding 95 percent confidence intervals. All *p* values were determined with two-tailed tests. A *p* value < 0.05 was considered as statistically significant.

## 3. Result

**Figure 1:**
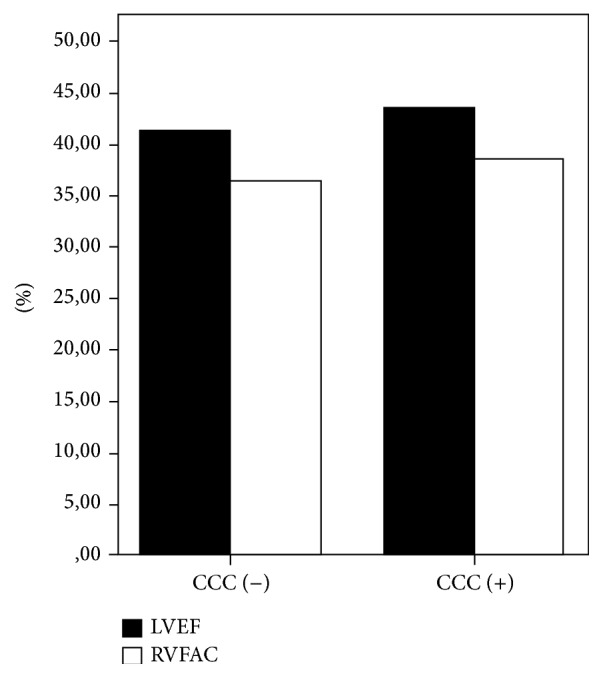
The difference of the groups in respect to left ventricular ejection fraction (LVEF) and right ventricular fractional area change (RVFAC).

There were 147 patients without CCC (mean age 53.5 ± 10.1 years, 81% men) and 88 patients with CCC (mean age 57.5 ± 10.5 years, 83% men). Baseline clinical, echocardiographic characteristics, beginning clinical values, and in-hospital therapy of the groups were listed in Table 1. There were no statistically significant differences in respect to age, sex, coronary risk factors, beginning clinical values, left ventricular ejection fraction (Figure 1), and in-hospital therapy among the groups. Echocardiographic RV parameters were significantly different between the two groups. RVFAC (Figure 1) and TAPSE were significantly lower in patients without CCC. Right ventricular free-wall index was significantly higher in patients without coronary CC.

Angiographic procedural data of the groups were listed in Table 2. There were no statistically significant differences between the groups in respect to presence of multivessel disease, TIMI flow before PCI in RCA, and major RV branch artery. Coronary lesion location was significantly different between the groups. A higher number of patients with proximal coronary lesion was observed in patients with CCC. There was no difference between the groups in respect to TIMI flow after PCI in main RCA and in major RV branch artery.

RVI was noted less frequently in patients with coronary CC than in those without coronary CC. In addition, the patients with coronary CC were less likely to have complete atrioventricular block, VT/VF, cardiogenic shock, and in-hospital death compared to those without coronary CC (Table 3).

Age, male sex, diabetes mellitus, left ventricular ejection fraction, door to balloon time, time from symptoms onset to PCI, absence of CCC to RCA, multivessel coronary artery disease, coronary lesion location, and successful primary angioplasty were analyzed using multivariate logistic regression to estimate their predictive value for in-hospital death (Table 4). Absence of coronary CC to RCA was found to be the independent predictor for in-hospital death among them (odds ratio 4.0, 95% CI 1.8–12.6; *p* = 0.03).

## 4. Discussion

In the present study, we found that the absence of CCC to the ischemic myocardium in the early hours of infarction was an independent predictor of mortality in patients with inferior STEMI undergoing PCI. Thus, the absence of CCC to the infarct-related artery may be considered an adverse risk factor in the assessment of short-term prognosis after inferior STEMI.

CCC may play an important role in maintaining viable myocardium after abrupt coronary occlusion, and it is a primary determinant of LV function recovery after late mechanical infarct artery reperfusion [10, 11]. However, the clinical impact of collateral flow on infarct size during acute myocardial infarction (AMI) is controversial. Elsman et al. showed the protective effect of collaterals on infarct size in patients with STEMI treated by primary intervention, but this study showed no protective effect on microvascular perfusion of collateral flow to acutely occluded nonleft anterior descending coronary artery related infarcts [12]. On the other hand, Antoniucci et al. found no protective effect of collaterals on infarct size in patients undergoing primary intervention within 6 hours of AMI onset [13]. As a result, there is a paucity of data regarding the pathophysiologic significance of collaterals observed during AMI and controversy exists regarding their clinical impact. Existing collaterals cannot prevent infarction in the majority of cases; however, they may limit the ongoing damage. In our study, the patients without CCC are approximately two times more likely to have RVI compared to those with CCC. This finding demonstrated that coronary CC may have provided some protection against the expansion of RV infarct size within 12 hours of AMI.

RVI is associated with other complications of RCA occlusion, including complete atrioventricular (AV) block, VT/VF, and cardiogenic shock [14, 15]. These complications with RVI are related to increased in-hospital mortality. In our study, patients with CCC had the lower incidence of complete atrioventricular block, VT/VF, and cardiogenic shock. Several studies have demonstrated that CCC at the time of AMI reduces infarct size; this effect might partially explain how CCC protect against RVI, AV block, and cardiogenic shock. In parallel to these findings, our study also showed that patients without CCC had significantly worse regional right ventricular free-wall motion, low TAPSE, and RVFAC indicating larger RV involvement. The lower incidence of AV block in patients with CCC could be partially explained by the interception of subsequent ischemic burden on the atrioventricular node due to operating collateral vessels present at the time of infarction since several studies have shown an association between complete heart block and overall infarct size [16, 17]. Furthermore, in our study, the incidence of combined VT/VF was lower in the patients with CCC than in those without CCC. One of several suggested protective mechanisms against VT/VF development is the reduction of ischaemia-induced prolongation of the QT interval which could potentially lead to fatal arrhythmias [18]. As a result, it can be hypothesized that the collateral circulation as a bridging network might reduce ischaemic burden on involved myocardium, ending with better clinical outcomes [18].

The lower incidence of cardiogenic shock in patients with CCC could be explained by the preservation of RV free-wall motion as long as operating collateral vessels are present at the time of infarction. This could ameliorate the systolic dysfunction and promote a partial functional recovery of the involved wall segment early after AMI. This mechanism can also be plausible for the observation of other studies dealing with left ventricular function [5, 19, 20]. Additionally, Waldecker et al. showed that the absence of collaterals was related to the early occurrence of cardiogenic shock in patients with inferior STEMI. However, in the literature, there has not been any clinical study reporting that operating collateral vessels prevent RV function early after AMI [21]. But an experimental study by Laster et al. reported that restoration of collateral perfusion facilitated late recovery of right ventricular free-wall function in closed-chest dogs [22]. So, coronary CC can prevent expansion of infarct size improving residual RV or LV ejection fraction and limiting further RV dysfunction early after AMI. In addition to the fatal arrhythmias, the other reasons for in-hospital mortality are cardiogenic shock due to RV dysfunction and complete AV block among patients with RV infarctions. Thus, it seems that protection of RV functions and AV node from coronary ischemia will reduce in-hospital mortality.

Although average left ventricle ejection fraction of the group with coronary collateral had tendency to be higher than the group without coronary collateral, the difference was not statistically significant. Small difference may be a result of improvement in right and left ventricle function due to presence of coronary collateral. Cardiogenic shock in the study was purely related to right ventricle infarction thus it cannot be attributed to left ventricle ejection fraction. And also the finding confirmed that presence of coronary collateral circulation improves right ventricle functions and reduces the frequency of cardiogenic shock.

In our study, we found that the incidence of in-hospital mortality was lower in the patients with CCC than in those without CCC. Pérez-Castellano et al. also found a significantly higher in-hospital mortality among patients without collateral vessels with acute anterior myocardial infarction during the six-hour time window after symptom [23]. Although controversial results have been reported on CCC in the literature, the mainstream of the data supports beneficial effect of CCC on clinical outcomes [24]. It seems that controversial findings stemmed from heterogeneity of study populations (clinical settings of acute and subacute myocardial infarction or stable coronary artery disease), difference of myocardial infarction types (mostly anterior wall), difference of methods or definitions used to assess collateral circulation, difference of applied treatment modalities (medical, PCI, or thrombolytic), and most importantly lack of differentiation of left-sided and right-sided collaterals in the relevant clinical setting [13, 24–26]. Thus, our study was unique in respect to homogeneity of study population, including only subject with inferior STEMI who has undergone primary PCI and assessing CCC as absent (Rentrop 0) or present (Rentrop 1, 2, or 3) rather than defining it as low or high collateralization. Additionally, our study enrolled only the patients with RCA-dependent inferior STEMI, contributing homogeneity of the population and allowing more precise and direct associations to be found. Thus, according to these results we can easily assume that CCC had significant role in clinical outcomes in the setting of inferior STEMI accompanying primary PCI.

## 5. Limitation

Angiographically detected collateral flow provides only an estimate of existing absolute collateral flow since only collaterals 100 *μ*m or more in diameter can be identified. Collateral flow can also be evaluated with methods such as myocardial contrast echocardiography, cardiac nuclear imaging, and pressure-derived collateral flow index with better quantification but indirectly [10, 27, 28]. However routine use of these methods in clinical practice is not feasible in the setting of acute myocardial infarction treated with PCI. Due to shortness of time, echocardiographic examination was performed following PCI; thus possibility of recovery in RV functions just after PCI and assessment of interobserver variability cannot be excluded. Additionally, the determination of collaterals was made after the AMI. Thus this study does not answer the “old” question whether collaterals before AMI limit the severity of the AMI.

## 6. Conclusion

Presence of angiographically detectable CCC was associated with better in-hospital outcomes including RVI, complete AV block, cardiogenic shock, and VT/VF in patients with inferior STEMI.

## Figures and Tables

**Table 1:** Baseline clinical, echocardiographic characteristics and in-hospital therapy.

Variable	Coronary collateral circulation		*p* value
	Absent (*n* = 147)	Present (*n* = 88)	
Age (years)	53.5 ± 10.1	57.5 ± 10.5	0.15
Male [*n* (%)]	119 (81)	71 (80)	0.90
Hypertension [*n* (%)]	64 (44)	37 (42)	0.82
Hyperlipidemia [*n* (%)]	45 (30)	23 (26)	0.35
Diabetes mellitus [*n* (%)]	42 (29)	20 (23)	0.25
Smoke [*n* (%)]	88 (60)	49 (56)	0.50
Family history of CAD [*n* (%)]	39 (27)	28 (32)	0.35
Previous MI [*n* (%)]	27 (18.4)	18 (20.5)	0.70
Time from symptoms onset to PCI (hours)	4.15 ± 1.36	4.25 ± 1.50	0.60
Door to balloon time (minutes)	65 ± 12.8	69.5 ± 13.2	0.17
Left ventricular ejection fraction (%)	41.3 ± 3.4	43.4 ± 2.9	0.08
Right ventricular fractional area change (%)	36.5 ± 4.6	38.7 ± 4.0	0.02
Tricuspid annular plane systolic			
Excursion (mm) (TAPSE)	15.6 ± 2.8	17.8 ± 2.2	0.01
Right ventricular free-wall index	1.5 ± 0.5	1.2 ± 0.6	0.006
In-hospital therapy			
Aspirin [*n* (%)]	140 (95.2)	86 (97.7)	0.35
ACEI-ARA [*n* (%)]	85 (58)	52 (59)	0.84
Clopidogrel [*n* (%)]	131 (89)	80 (91)	0.60
Ticagrelor [*n* (%)]	16 (11)	8 (9)	0.66
Statin [*n* (%)]	141 (96)	86 (98)	0.46
Glycoprotein IIb/IIIa inhibitor [*n* (%)]	18 (12.2)	13 (14.8)	0.58

Data are expressed as mean ± SD for normally distributed data or count (percentage) for categorical variables; ACEI-ARA: angiotensin-converting enzyme inhibitor-angiotensin II receptor antagonist; CABG: coronary artery by-pass graft; CAD: coronary artery disease; IACE: in-hospital adverse clinical events; MI: myocardial infarction; PCI: percutaneous coronary intervention.

**Table 2:** Preprocedural and postprocedural angiographic characteristics related to coronary collateral circulation.

Variable	Coronary collateral circulation		*p* value
	Absent (*n* = 147)	Present (*n* = 88)	
*Preprocedural*			
Number of diseased coronary arteries			0.28
1 [*n* (%)]	83 (56.4)	43 (48.8)	
2 [*n* (%)]	31 (21.8)	18 (20.4)	
3 [*n* (%)]	33 (22.4)	27 (30.7)	
Coronary lesion location at RCA			0.006
Proximal [*n* (%)]	75 (51)	61 (69.3)	
Distal [*n* (%)]	72 (49)	27 (30.7)	
TIMI flow before PCI in main RCA			0.8
TIMI 0 [*n* (%)]	131 (89)	79 (90)	
TIMI 1 [*n* (%)]	16 (11)	9 (13)	
TIMI flow before PCI in major RV branch artery			0.36
TIMI 0-1 [*n* (%)]	87 (59.2)	50 (56.8)	
TIMI 2-3 [*n* (%)]	60 (40.8)	38 (43.2)	
*Postprocedural*			
TIMI flow after PCI in main RCA			0.18
Unsuccessful (TIMI 0-1) [*n* (%)]	19 (13)	10 (11.3)	
Successful (TIMI 2-3) [*n* (%)]	128 (87)	78 (88.6)	
TIMI flow after PCI in major RV branch artery			0.36
Unsuccessful (TIMI 0-1) [*n* (%)]	15 (10.2)	7 (8)	
Successful (TIMI 2-3) [*n* (%)]	132 (89.8)	81 (92)	

PCI: percutaneous coronary intervention; RCA: right coronary artery; RV: right ventricular; TIMI: thrombolysis in myocardial infarction.

**Table 3:** Clinical outcome according to presence and absence of CCC.

Outcome	CCC		*p* value
	Absent	Present	
	*n* = 147	*n* = 88	
RV infarction [*n* (%)]	46 (31.2)	16 (18.2)	0.007
Cardiogenic shock [*n* (%)]	22 (15)	9 (10.2)	0.005
Complete AV block [*n* (%)]	28 (19)	6 (6.8)	0.01
VT/VF [*n* (%)]	25 (17)	5 (5.7)	0.01
Death [*n* (%)]	15 (10.2)	2 (2.3)	0.02

AV: atrioventricular; CCC: coronary collateral circulation; RV: right ventricular; VT/VF: ventricular tachycardia/ventricular fibrillation.

**Table 4:** Univariate and multivariate logistic regression analysis for in-hospital death.

Variable	Univariate			Multivariate		
	Unadjusted	95% CI	*p* value	Adjusted	95% CI	*p* value
	OR			OR		
Age	1.05	1.02–1.08	0.01	1.03	1.03–1.07	0.04
Male sex	0.7	0.4–1.2	0.25			
Diabetes mellitus	1.7	0.6–3.20	0.53			
Left ventricular ejection fraction	1.3	0.75–3.1	0.42			
Door to balloon time	1.03	1.03–1.07	0.04	1.02	1.04–1.06	0.05
Time from symptoms onset to PCI	1.07	0.64–1.80	0.80			
Absence of CCC to RCA	4.4	1.5–14.5	0.01	4.0	1.8–12.6	0.03
Multivessel coronary disease	1.8	0.94–3.2	0.08			
Coronary lesion location	3.0	1.2–7.8	0.03			
Successful primary angioplasty	0.6	0.20–3.10	0.20			

CI: confidence interval; OR: odds ratio; PCI: percutaneous coronary intervention; RCA: right coronary artery.
